# Development of a Predictive Dashboard With Prescriptive Decision Support for Falls Prevention in Residential Aged Care: User-Centered Design Approach

**DOI:** 10.2196/63609

**Published:** 2025-04-07

**Authors:** S Sandun Malpriya Silva, Nasir Wabe, Amy D Nguyen, Karla Seaman, Guogui Huang, Laura Dodds, Isabelle Meulenbroeks, Crisostomo Ibarra Mercado, Johanna I Westbrook

**Affiliations:** 1 Centre for Health Systems and Safety Research Australian Institute of Health Innovation Macquarie University North Ryde, NSW Australia; 2 The MARCS Institute for Brain, Behaviour and Development Western Sydney University Sydney, New South Wales Australia

**Keywords:** falls prevention, dashboard architecture, predictive, sustainability, challenges, decision support, falls, aged care, geriatric, older adults, economic burden, prevention, electronic health record, EHR, intervention, decision-making, patient safety, risks, older people, monitoring

## Abstract

**Background:**

Falls are a prevalent and serious health condition among older people in residential aged care facilities, causing significant health and economic burdens. However, the likelihood of future falls can be predicted, and thus, falls can be prevented if appropriate prevention programs are implemented. Current fall prevention programs in residential aged care facilities rely on risk screening tools with suboptimal predictive performance, leading to significant concerns regarding resident safety.

**Objective:**

This study aimed to develop a predictive, dynamic dashboard to identify residents at risk of falls with associated decision support. This paper provides an overview of the technical process, including the challenges faced and the strategies used to overcome them during the development of the dashboard.

**Methods:**

A predictive dashboard was co-designed with a major residential aged care partner in New South Wales, Australia. Data from resident profiles, daily medications, fall incidents, and fall risk assessments were used. A dynamic fall risk prediction model and personalized rule-based fall prevention recommendations were embedded in the dashboard. The data ingestion process into the dashboard was designed to mitigate the impact of underlying data system changes. This approach aims to ensure resilience against alterations in the data systems.

**Results:**

The dashboard was developed using Microsoft Power BI and advanced R programming by linking data silos. It includes dashboard views for those managing facilities and for those caring for residents. Data drill-through functionality was used to navigate through different dashboard views. Resident-level change in daily risk of falling and risk factors and timely evidence-based recommendations were output to prevent falls and enhance prescriptive decision support.

**Conclusions:**

This study emphasizes the significance of a sustainable dashboard architecture and how to overcome the challenges faced when developing a dashboard amid underlying data system changes. The development process used an iterative dashboard co-design process, ensuring the successful implementation of knowledge into practice. Future research will focus on the implementation and evaluation of the dashboard’s impact on health processes and economic outcomes.

**International Registered Report Identifier (IRRID):**

RR2-https://doi.org/10.1136/bmjopen-2021-048657

## Introduction

Falls are the second leading cause of unintentional injury deaths worldwide, with those older than 60 years having the highest risk of death or serious injury [1]. People living in residential aged care facilities (RACFs; ie, nursing homes and care homes) are three times more likely to fall than their community-dwelling peers and are ten times more likely to sustain fall-related injuries [2]. Studies have found that falls are often predictable and preventable and that the health and economic burden of falls could be minimized if effective prevention strategies are implemented [3-5].

Falls prevention is an issue of major importance in RACFs. Various fall risk assessment tools (FRATs) are used by RACF staff internally to identify residents at greatest risk of falls [6]. However, when assessed, the predictive performance of these tools has generally shown to be poor [6,7]. Moreover, FRATs are most often completed when residents are admitted to a facility or after a fall incident, and thus, only represent data collected at a single point in time, resulting in a static prediction that does not reflect changing falls risk factors (eg, if a resident’s medication use changes) [6].

As a growing number of RACFs implement electronic health record (EHR) systems, new opportunities have emerged to develop a personalized, dynamic approach to predicting residents’ fall risk by taking advantage of multiple potential contributory factors [8]. Some studies have integrated routinely collected EHR data including vital signs into the development of fall prediction tools through the application of machine learning models [9]. Despite these tools exhibiting superior performance in contrast to conventional FRATs, the risk predictions generated are often difficult to interpret and apply in practice. Thus, relying solely on these models will not enhance decision-making to provide a personalized care strategy for fall prevention and management. Health dashboards that can integrate these predictive models in combination with visualization features may be a more effective intervention for supporting staff in RACFs.

A dashboard can be defined as an at-a-glance real-time or near real-time processing interface, showing a graphical presentation of the current or recent status, along with historical trends or organizational key performance indicators to support decision-making [10]. There have been some attempts to develop a dashboard for fall prevention purposes globally. The falls dashboard, developed by the Network of Patient Safety Databases and managed by the US Department of Health and Human Services, collects nonidentifiable patient-related fall incident data from health care service providers. These data are then aggregated to a national level, analyzed, and visualized using the Network of Patient Safety Database dashboards and chartbooks to identify and track patient safety risks nationally [11]. Similarly, the New Zealand Health Quality and Safety Commission has established a falls and fracture outcomes dashboard to help the health sector evaluate the benefits of the services provided to older people [12]. Such aggregated dashboards are helpful for monitoring, although they offer limited assistance in predicting and preventing falls in routine care settings. To the best of our knowledge, no previous study has reported the development of a dashboard designed to predict fall risks for individual residents and aid decision-making in preventing and managing falls. As part of a comprehensive research program aimed at enhancing fall prevention and the well-being of older adults in RACFs [8], this paper details the design and development process of a predictive analytics dashboard with prescriptive decision support for fall risk prediction, prevention, and management. We discuss the challenges encountered and the strategies used to overcome them during its development.

## Methods

### Participants

This project was conducted in collaboration with a large, aged care provider that operates 24 RACFs in Sydney, Australia. The development process of the dashboard spanned from January 1, 2022, to December 31, 2023. For the dashboard views and study sample, this study used data from both respite and permanent residents who were present in a RACF between January 1, 2021, and December 31, 2021. The dashboard was designed using participant data that was extracted onto Excel sheets from the provider. After the development, the dashboard was connected to the provider’s live data sources through a data lake.

### Ethical Considerations

All methods and analyses in this study were conducted in accordance with the principles of the Declaration of Helsinki. The study received ethics approval from the Macquarie University Human Research Ethics Committee (52019614412614). As a retrospective cohort study using deidentified, routinely collected aged care data, a waiver of informed consent was granted by the committee, in line with the Australian National Statement on Ethical Conduct in Human Research (Sect. 3.3.14(a)), which allows for the use of previously collected deidentified data.

### Dashboard Development Process

#### Overview

**Figure 1:**
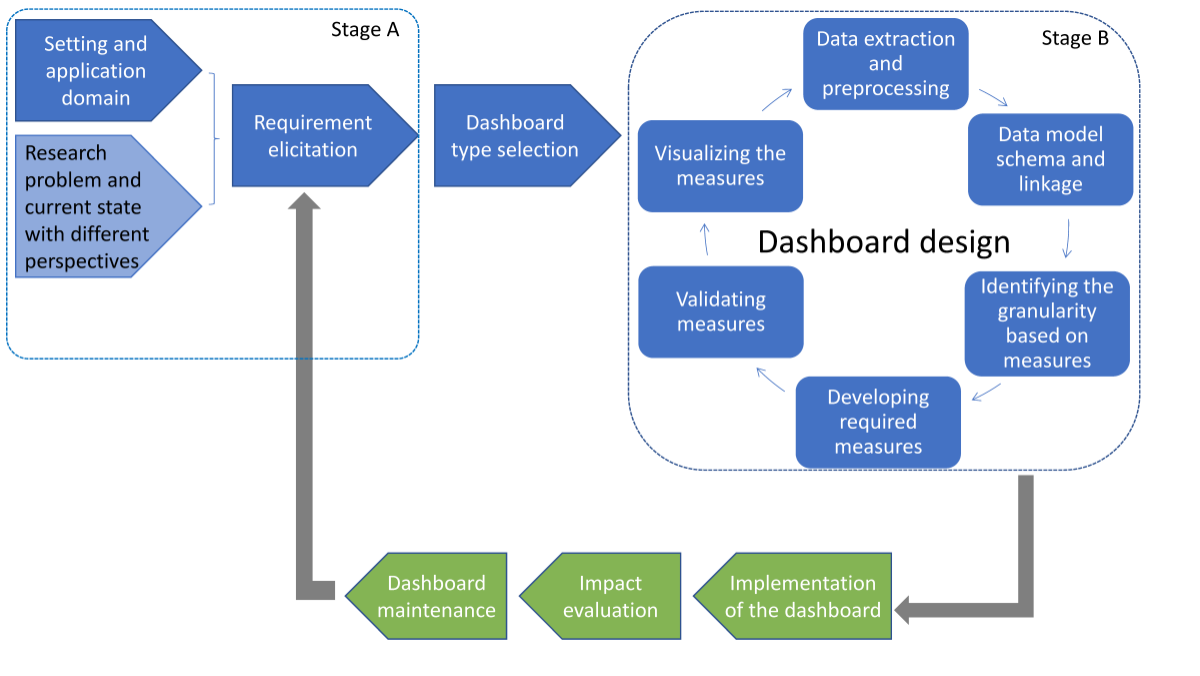
The dashboard development process.

We followed an iterative process to co-design the dashboard, with intended dashboard end users (ie, RACF staff, general practitioners, and consumers at RACFs), along with domain experts in the field of falls prevention and management. Figure 1 highlights the dashboard development process followed during the study. The stakeholders were involved continuously in Stage A and Stage B through regular discussions, interviews, and workshops, which will be described in detail elsewhere. The steps colored in blue are described in this paper and the final steps relating to implementation and evaluation colored in green are in progress and will be reported in subsequent papers.

#### Setting and Application Domain

Domain knowledge can be defined as the knowledge about the environment in which the proposed system will operate [13]. Domain knowledge was obtained from the aged care provider through discussions and analysis of data related to falls incidents and the Peninsula Health Falls Risk Assessment Tool (PH-FRAT) risk assessments (ie, to determine the current falls rate across facilities, the characteristics of falls incidents and contributing factors listed in falls incidents). PH-FRAT was developed in 1999 by Peninsula Health in Victoria, Australia, and is a validated and user-friendly tool for screening and assessing fall risk, as well as managing strategies to reduce this risk [14]. Information about predictors of falls and strategies used to prevent falls in the RACFs was obtained by systematically reviewing published literature on predictive models for fall prevention, and the effectiveness of fall prevention interventions [15,16]. The identified predictors of falls in older people have been incorporated into the development of the embedded predictive model, which is reported elsewhere [17].

#### Research Problem and Current State With Different Perspectives

Considering the suboptimal predictive performance of current tools [6], there is a need for a dashboard embedded with a dynamic predictive model and decision support (research problem). Moreover, falls prevention and management requires action at different levels of an organization’s hierarchy from management to clinical staff. Therefore, it was important to gather different perspectives. For this purpose, a series of discussions were held between researchers and RACF stakeholders across a 2-stage process (Figure 1). In Stage A discussions, different perspectives on falls prevention and management in RACFs were gathered while identifying the dashboard stakeholders and their information requirements. In Stage B, dashboard stakeholders engaged in rounds of iterative feedback workshops to support the dashboard designing process. A qualitative analysis of these interviews will be reported elsewhere.

#### Requirement Elicitation

The requirement elicitation stage was based on three fundamental tasks: (1) identifying the individual stakeholders; (2) eliciting stakeholder requirements depending on the role of the stakeholder; and (3) integrating, refining, and organizing the requirements identified [18].

In Stage A, baseline interviews were conducted to understand the current utilization of health care data and information by potential dashboard end users. The aim was to identify the information requirements essential for enhancing decision-making in a clinical dashboard. In this process, the individual stakeholders and information providers of the dashboard were identified along with their high-level user expectations [10]. This also facilitated a precise understanding of the individuals with different perspectives of falls prevention and management, who would be accessing each dashboard view. The information collected from these interviews was used to define measures that should be generated in the dashboard backend.

From these identified measures, the designers then evaluated the variability of information and organized the information requirements based on the level of reporting in the dashboard (ie, resident level, facility level, or organizational level). This further supported the dashboard design stage in arranging visual plots and tables within views.

#### Dashboard Type Selection

Power BI (PBI; Microsoft Corp) was selected on the grounds of digital data visualization, easy implementation at the user end, and stable R programming integration for advanced statistical analysis and plots. In this step, the initial prototype of the dashboard was created and shared with our partner organization and refined through collaborative efforts.

#### Dashboard Design

##### Overview

The aim of this step was to meet the user requirements for preventing and managing falls by effectively presenting resident data and fall risk information to support informed decision-making. As in Figure 1, this step included an iterative process that included data extraction, preprocessing, and linkage along with identification of the granularity of data, development, validation, and visualization of the measures on the front end.

##### Data Extraction and Preprocessing

Access to four datasets was obtained: the resident profile (includes residents’ demographics and admission information); all medications administered to residents; the organization’s PH-FRATs (this aged care providers used the PH-FRAT) to obtain information related to falls risk assessments; and incident dataset (includes information related to all fall incidents and pressure injuries; Table S1 in Multimedia Appendix 1).

The profile dataset included a free text field that reported the comorbidities along with other special needs of the patient at admission (ie, health status). From this field, comorbidities present at admission were identified using the R-programmed version of the “aged care health status algorithm” [19] within PBI. The algorithm identifies the health conditions using free text fields from EHRs [20]. All medications in the dataset were coded using the Anatomical Therapeutic Chemical codes [21]. These datasets were then linked in the dashboard backend.

The extracted datasets for the study period underwent an external analysis for this study using R programming language (version 4.3.3; R Core Team) and were also subsequently used for its intended purpose within PBI. A descriptive analysis of the admission-related information from the resident profile dataset is reported appropriately (Table S2 in Multimedia Appendix 1). Resident characteristics from the resident profile dataset, FRAT dataset, and daily medication administration data are recorded in Table S3 in Multimedia Appendix 1.

##### Data Model Schema and Linkage

**Figure 2:**
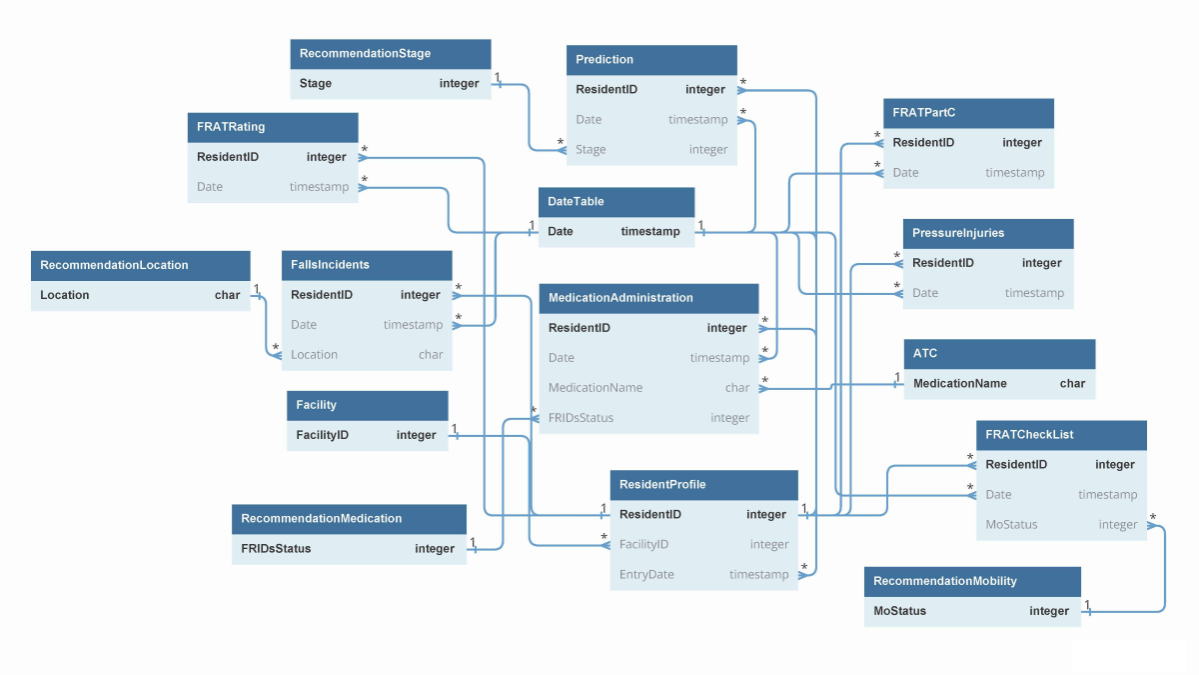
Simplified view of the data model schema. ATC: Anatomical Therapeutic Chemical code; FRAT: falls risk assessment tool.

A star schema was adopted as much as possible where the fact table is centered and surrounded by the multiple dimension tables. The resident profile dataset was used as the main fact table that contains quantitative information (facts) about the residents [22]. Other datasets were used as dimension tables, which describe the events that residents experience during their stay [22]. The data tables were then linked as presented in the simplified form of the database schema as shown in Figure 2. The real schema on the backend of the dashboard is an extension of this and is more advanced.

##### Identifying Granularity Based on Measures

To determine the granularity of the data tables, we considered the dashboard end users, information requirements (measures), and level of reporting that were preidentified. This step was done in parallel or a back-and-forth manner with the data model schema (ie, preceding step) and development of required measures (ie, proceeding step). A higher granularity was preferred for most of the datasets to maintain sustainable analytical power to answer new user requirements within the designing stage [23]. Subsequently, the data tables were transformed to simply calculate the measures, or measure calculations were adjusted in a way to meet the form and granularity in the extracted tables. In certain instances, highly granular data tables were summarized to a lower-level granularity to support the prediction model and avoid intricate calculations during measure development.

##### Developing the Required Measures

In this step, measures were calculated on the transformed tables using Data Analysis Expression (DAX). Measures related to some figures such as funnel plots were created using embedded R programming in the PBI backend.

##### Validating the Measures

To ensure the accuracy and reliability of the information presented, the calculated measures were validated with provider-extracted datasets. The user input filters on each view were also tested during the validation process to identify whether there were any errors. If errors were identified in the data, remedial action was taken after a thorough examination of the underlying causes, in data extraction, data linkage, data preprocessing, or DAX formula.

##### Visualizing the Measures

The calculated measures were appropriately visualized using statistical plots, tables, and responsive figures on the dashboard, which comprises several dashboard views intended for various users at different levels in the hierarchy. The above-mentioned steps within the dashboard design stage were enclosed in a co-design process as explained below.

### Dashboard Co-Design and Work Process Features (Stage B)

The core constructs of the integrated Promoting Action on Research Implementation in Health Services [24] framework were considered to facilitate innovation in the development of the dashboard: (1) innovation refers to the alignment of new insights including the predictive risk of falls captured by the dashboard with the current priorities and practices for falls prevention at RACFs, (2) recipients refer to the RACFs staff and other external stakeholders who will be affected by and influence the implementation of the dashboard, (3) context refers to the RACFs including the organization (inner layer) and wider health system (outer layer) around falls prevention and management, and (4) facilitation refers to the process that activates the implementation through assessing the characteristics of the three previously mentioned constructs.

The core constructs were integrated into the dashboard development process and the design was meticulously refined through a series of workshops (ie, facilitation) involving diverse teams of experts from various layers. These teams included general practitioners, geriatricians, allied health staff from RACFs (including nurses and clinical care managers), and human-computer interaction experts (ie, recipients). The collaborative workshops aimed to identify and implement enhancements to the dashboard, ensuring that it caters to the needs and preferences of all relevant stakeholders (ie, innovation). The design features (eg, color, graph types, positioning, filters, slices, and data views) and content for decision-making (ie, information included or missing on the dashboard and predictive model output) were considered. In these workshops, the development stage prototypes were presented to gather information on decision-making on client care and to identify the dashboard views that required improvements in managing falls. Current priorities and practices involved in managing falls at RACFs (ie, context) were also identified.

### Descriptive and Diagnostic Analytics: Measures, Indicators, and Statistical Tests

All falls, injurious falls, and falls requiring hospitalization were identified as the main indicators in the dashboard. The total number of falls, the crude incident rate (ie, falls rate per 1000 resident days), the percentage of injurious falls, and the percentage of hospitalization were calculated for each outcome measure to enhance the decision support capability at an organizational level.

Funnel plots were used to visualize crude incident rates with 95% and 99% control limits, relative to the number of admissions. A time series plot was used to visualize the percentage of injurious falls and hospitalization due to falls.

### Predictive Analytics With Resident Tailored Recommendations: Dynamic Falls Predictive and Monitoring Model

A dynamic predictive tool was developed to classify each resident’s risk of falls using a technique called stratified landmarking and reported in detail elsewhere [17]. Initially, 116 variables were screened, and the most relevant variables on the fall outcome were selected using Collett’s [25] variable selection approach. Two separate models based on dementia status were developed [17]. The final two models were based on patient demographics, dementia status, fall risk–increasing comorbidities such as cerebrovascular accident, visual impairment, osteoporosis or fracture, medication use, falls history, other risk factors such as psychological status, mobility status, behavior status, and activities of daily living [17].

The predictive model used in this study was developed and internally validated using retrospective data from the same provider [17]. The scores based on model coefficients and baseline hazards were then embedded into the dashboard backend and calculated the resident’s probability of falling in a near real-time manner. The predictions generated by the model are updated every 24 hours given the modifiable risk factors such as medication use, previous fall incidents, and comorbidities.

As mentioned in the introduction, relying solely on a predictive model will not improve decision-making for developing a personalized care strategy for fall prevention and management. Additionally, the dashboard that visualizes risk factors at both the resident and facility levels should be used to enhance the interpretability of these predictions and other associated risk factors of falls. This dashboard visualizes the risk factors for falls, along with changes in risk and risk stages derived from the predictive model. Furthermore, to offer a more prescriptive approach based on these predictions, evidence-based, resident-tailored recommendations have been developed as follows.

Resident-tailored recommendations are generated using a rule-based system in the dashboard’s backend. The system identifies residents’ mobility issues from PH-FRAT data and detects the use of fall risk–increasing drugs (FRIDs) from daily medication records. Fall incident data are used to determine the most frequent fall locations, while a predictive model assesses the current fall risk. Based on these factors, rule-based recommendations [26-28] are created and displayed in the resident fall view (Table S4 in Multimedia Appendix 1). Additionally, strategies identified through clinical judgment in the PH-FRATs are also displayed to enhance the personalization.

## Results

### Descriptive Profile of the Aged Care Provider, Facilities, and Residents

The aged care provider managed 24 RACFs. During the period of dashboard development, these RACFs housed 3686 resident admissions from 3057 unique residents. The median facility size was 124 (IQR 86-160) residents. Of the resident admissions, 71% (n=2622) were permanent. The median length of stay was 373 (IQR 41-1266) days (Tables S1 and S2 in Multimedia Appendix 1).

### Dashboard Evolution Within the Design Stage

The Stage A interviews and Stage B workshops identified themes and findings related to content, design, functionality, and decision support. This led the dashboard to evolve through a series of iterations (Multimedia Appendix 2). Two dashboard views namely, organizational falls and facility falls were designed for organizational managers, while another two views were designed for care staff and focused on residents, resident falls, and resident-level medications in detail. The intervention recommendations gathered on fall prevention and management from published randomized controlled trials, government reports, systematic reviews, and meta-analyses [26-28] are included in the recommendation panel for education purposes.

### Measures Developed in Dashboard Views

Textbox 1 presents the measures developed for each dashboard view. Given the interrelatedness of these views, certain measures used at the organizational level (such as total falls and falls rate [per 1000 resident days]) were also included at the facility level, while certain measures used at the resident level (ie, FRIDs use in last three days and polypharmacy indicator) were included in the medications in detail view.

**Measures available in the dashboard.**

**Organizational falls**

- Total falls
- Total falls requiring hospitalization
- Total injurious falls
- Falls rate (per 1000 resident days)
- Falls requiring hospitalization rate (per 1000 resident days)
- Injurious falls rate (per 1000 resident days)
- Number of admissions per selected period
- Resident days per selected period

**Resident falls**

- Current falls risk
- Current risk category
- Daily percentage change in risk
- Falls in last 6 months
- Fall risk–increasing drugs (FRIDs) use in the last three days
- Change in FRIDs use in last three days
- Polypharmacy indicator
- Antipsychotic indicator
- Recent falls risk assessment tool (FRAT) assessment date
- Recent cognitive status identified by Peninsula Health-FRAT
- Recent psychological status

**Facility falls**

- Total number of residents
- Residents having stage 1 pressure injury
- Residents having stage 2 pressure injury
- Residents having stage 3 pressure injury
- Residents having stage 4 pressure injury
- Residents having unstageable pressure injuries
- Residents having deep tissue pressure injuries
- Residents with a pressure injury

**Medications in detail**

- Polypharmacy indicator – without pro re nata and short course
- Daily FRIDs indicator
- Daily change in FRIDs
- Daily change in opioid use
- Daily change in anxiolytics
- Daily change in hypnotic and sedatives
- Daily change in beta blocking agents
- Daily change in vasodilators
- Daily change in antidepressants

### Dashboard Architecture

**Figure 3:**
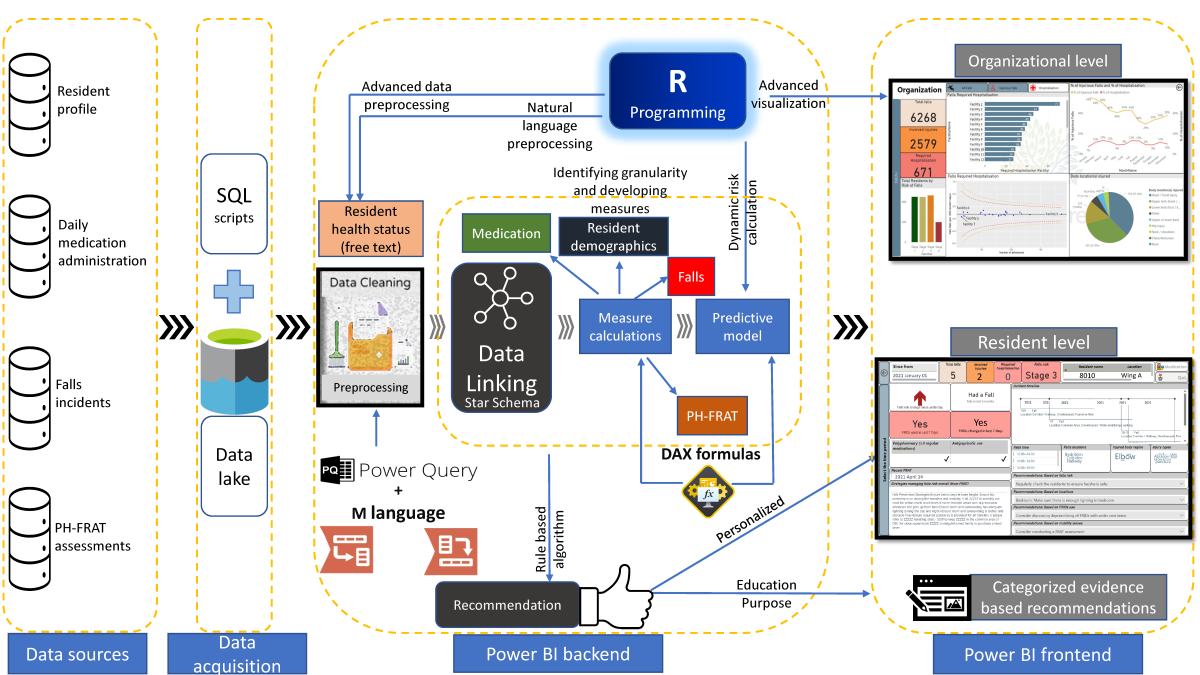
The dashboard architecture with four layers. DAX: Data Analysis Expression; PH-FRAT: Peninsula Health falls risk assessment tool.

Data flows through 4 layers, to formulate the necessary information (Figure 3).

The first layer includes the data sources where the aged care provider collects and stores data. The next layer is the data acquisition layer. It includes data extraction scripts such as SQL scripts, which collect data from the previous layer. As the dashboard was developed without a direct connection to the above data silos (sources), the data extractions at the beginning were done using SQL scripts for each data silo, including the original variable names and formats. This layer is also helpful in maintaining the reliability of the dashboard when underlying IT systems change by storing the original form of data that is compatible with the dashboard backend, which is the next layer. At implementation, this layer will be replaced with a data lake that feeds provider-level data to the dashboard backend.

Then the data extracted from the data lake are preprocessed and linked at the backend of the dashboard using the data model schema discussed (Figure 2). Previously identified measures and indicators are developed in this layer, to support the visualization of data at the front end of the dashboard. For this purpose, we used power query M language, DAX, and R programming. Risk calculations of the prediction model were carried out in this stage using R programming.

In the fourth layer, the information requirement of stakeholders is satisfied by visualization and reporting. The front end of the dashboard includes five views namely organizational falls, facility falls, resident falls, medications in detail, and recommendation panel. The filters are applied to various dashboard views depending on user needs as identified during the requirement elicitation and dashboard design stages. Moreover, statistical plots are used to enhance valid comparisons of indicators between facilities and to support decision-making.

### Dashboard View: Organizational Falls and Facility Falls

The organizational falls and facility falls are accessible by organizational and facility managers. Please note that some information about these views has been removed or modified to deidentify the residents and facility details.

**Figure 4:**
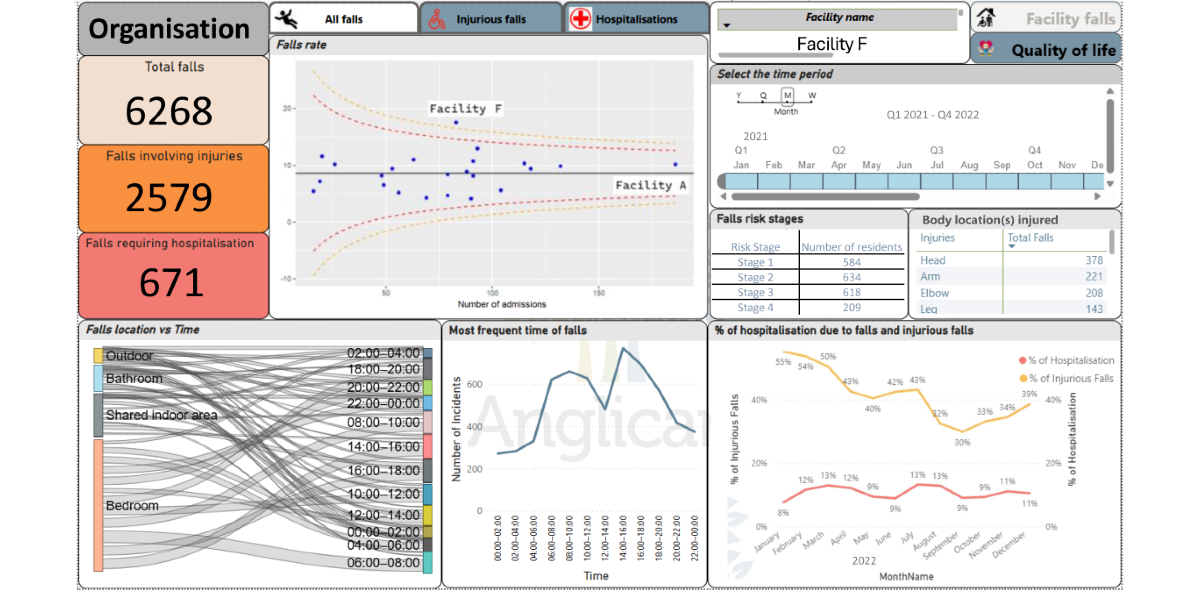
Organizational falls view.

The organizational falls view (Figure 4) includes organizational and facility-level information relevant to fall incidence. The time period and facility names are included as the filters. The information on this view can be updated with the values selected for these filters. The total number of falls along with injuries, and falls that required hospitalization are reported at the organizational level. The funnel plot in the view visualizes the crude fall rate. Users can visualize this plot for the three outcome measures namely all falls, injurious falls, and falls-related hospitalization. Funnel plots with the crude incident rates for each outcome measure enable valid comparisons between the facilities, as it can identify the outlying facilities with higher fall rates related to the number of admissions during the study period. The time series plot with a percentage of injurious falls and a percentage of hospitalization enables the user to investigate overall patterns of falls occurring in selected facilities or at an organizational level. This feature is helpful in identifying the longitudinal trend of injurious falls and hospitalization due to falls in facilities. The time series plot with the most frequent time of falls enables the user to investigate the time pattern of fall incidents. The most frequent locations of falls are linked with the time of falls by a Sankey plot. These plots are dynamic and responsive to the facility selected from the filters. Furthermore, the table with fall risk stages indicates the number of residents within each risk classification predicted by the embedded predictive model. The most frequent injured body locations are also reported in a table.

**Figure 5:**
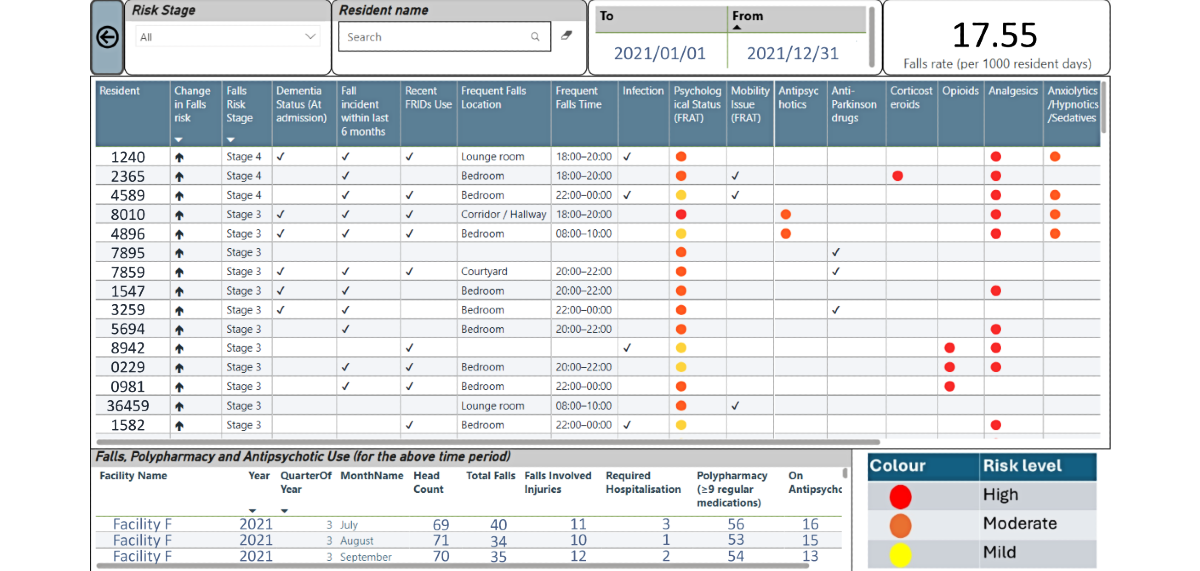
Facility falls view. FRAT: falls risk assessment tool.

The facility falls view (Figure 5) provides more details of the facility-specific falls along with facility characteristics. This view can also be accessed through the organizational level view using the “drill through” functionality in PBI. This view highlights the facility-specific fall rate per 1000 resident days for a specified time. To enhance the interpretability of the predictive model, resident-specific risk factors extracted from the model are reported in this view. Importantly, residents classified under each risk classification along with changes in fall risk compared to the previous day can be accessed in this view for effective fall prevention and management. The view also identifies comorbidities that increase fall risk (eg, dementia) and modifiable risk factors (eg, recent falls within the last six months, frequent locations and times of falls, mobility issues, psychological status, and the use of antipsychotic, opioid, and analgesic medications), all color-coded based on the level of risk identified by the predictive model. Additionally, facility-specific monthly indicators including four national mandatory quality indicators namely the percentage of care recipients: who experienced one or more falls; who were prescribed nine or more medications; who received antipsychotic medications; and with pressure injuries are also reported in this view. This view will also be included in a daily email notification to users once the dashboard is implemented.

### Dashboard View: Resident Falls and the Detailed Medications

Care staff will have access to the resident falls view, which includes the time period, facility name, falls risk classification (ie, derived from the predictive model), and resident identifier as filters.

**Figure 6:**
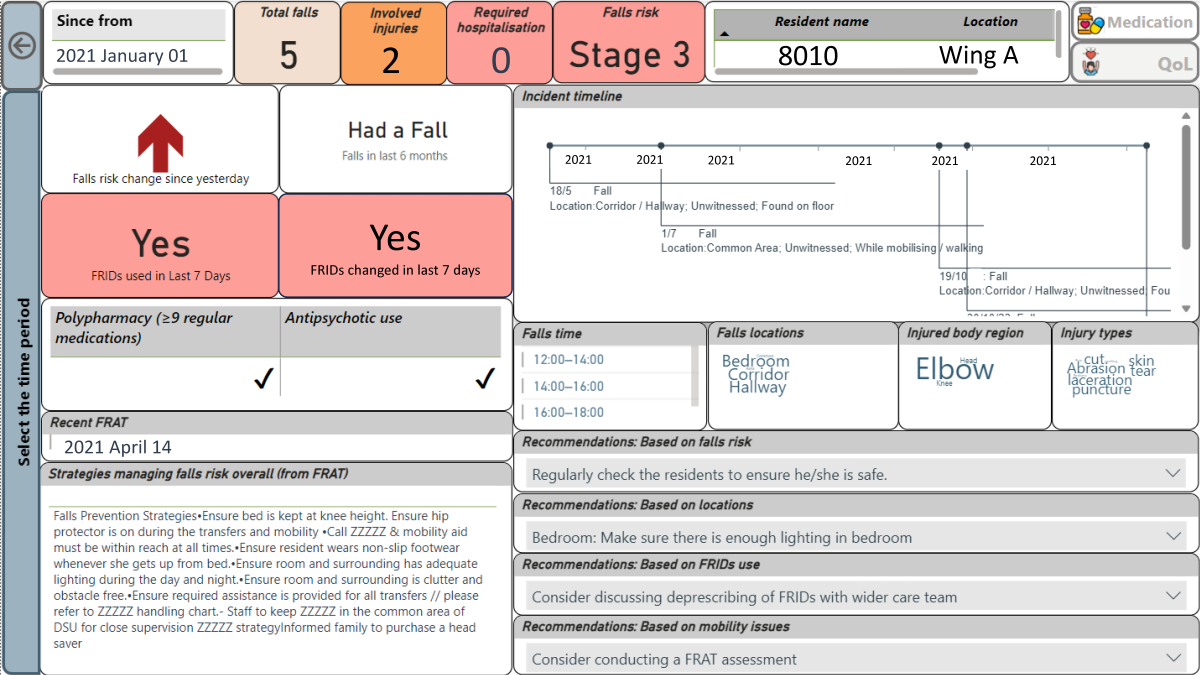
Resident falls view. FRAT: falls risk assessment tool; FRID: fall risk–increasing drug.

The resident falls view focuses on the resident-specific falls information along with recommendations to prevent falls (Figure 6). This can also be accessed using the facility falls view using drill-through functionality in PBI. The polypharmacy, FRIDs, and antipsychotic medication use are identified using the resident’s daily medication administration data. This view presents the current risk category along with the total number of falls faced by the resident during their stay. Risk classification derived from the predictive model is shown using color codes (ie, green to red) and text (ie, stages 1 to 4). Most importantly, the number of falls faced within the last 6 months is shown in this view. Patient-specific fall history is also visualized using a timeline along with the details of the events (ie, event type, injury status, and witnessed or unwitnessed). The sequence and the gaps between the fall incidents are highlighted in this plot. Furthermore, the word clouds visualize the injured body regions, injury types, locations, and time of the falls. At the bottom of the view, personalized recommendations are provided to the resident, which aligns with falls risk categorization, falls history, medication use, and mobility issues.

**Figure 7:**
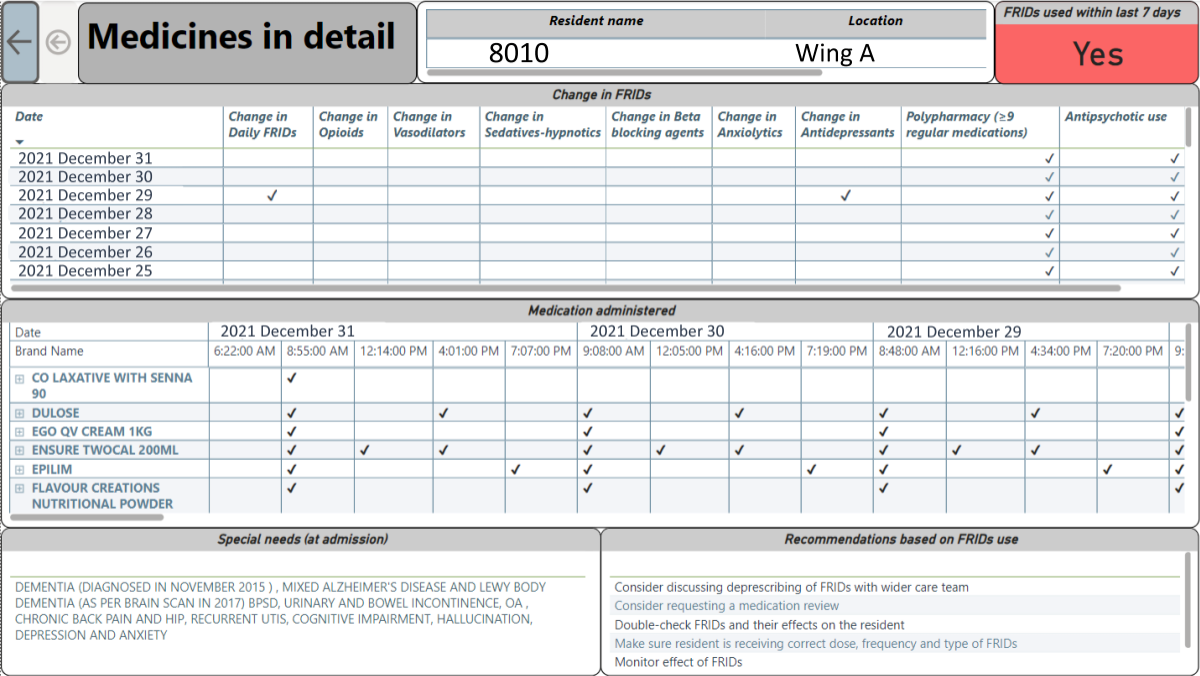
Medicines in detail. FRID: fall risk–increasing drug.

The medications in detail view (Figure 7) focuses on the resident-specific daily medication use. This view can be assessed using the “drill through” functionality enabled in the resident falls view*.* Here, changes in FRIDs (ie, whether the resident has previously used the drug, whether a medication was omitted, or a new drug added to the medication profile) within the last three days are identified and displayed, along with polypharmacy and antipsychotic medication indicators. The resident’s use of FRIDs is highlighted at the top. Daily medication administration records can also be accessed using this view. The special needs (ie, comorbidities and allergies) identified at the admission are included in the view to highlight the comorbidities, special needs, or risk factors of the resident.

### Dashboard View: Recommendation Panel

**Figure 8:**
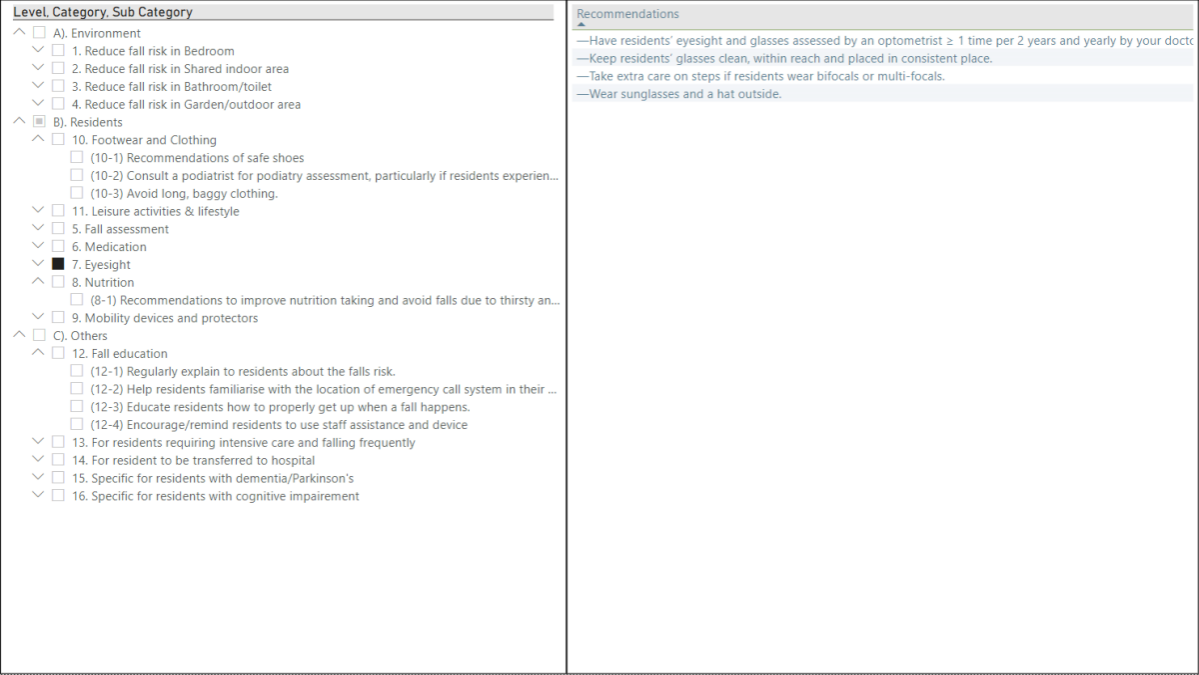
Recommendation panel.

As falls are affected by intrinsic and extrinsic factors [29], recommendations are grouped and reported in the recommendations panel (Figure 8) in three groups namely: resident (intrinsic), environment (extrinsic), and others followed by subcategorizations.

### Illustrative Use Scenario

#### Organizational Managers

A total of 6268 fall incidents were reported from the 3057 unique residents (729,410 resident days; Table S3 in Multimedia Appendix 1) between January 1, 2021, and December 31, 2021 (Figure 4). Of these, 2579 (41%) falls were injurious and 671 (11%) falls required hospitalization. The average fall rate was 8.36 per 1000 resident days for the study period (funnel plot). The percentage of injurious falls shows an overall gradual decline from 56% to 38%, whereas the percentage of falls requiring hospitalization is stable between 7% and 13% between January 1, 2021, and December 31, 2021. The majority of falls occurred in residents’ rooms. Injurious falls most often involved injuries to the head and face followed by upper limbs. When considering all falls, Facility A reported the highest number of falls. Facility F had the sixth-highest number of falls. However, the rate was above the 99% upper control limit (funnel plot), indicating a higher fall rate (per 1000 resident days) compared to other facilities with a similar number of admissions. Therefore, organizational managers can prioritize Facility F in intervening. They also can select Facility F and drill through to the facility falls view for a more detailed investigation.

#### Facility Managers

A total of 83 admissions were reported for facility F during the study period, with a fall rate of 17.55 per 1000 resident days (Figure 5). As of December 31, 2021, 3 (4%) residents were categorized with a fall risk of stage 4, while 13 (18%) residents were categorized with a risk of stage 3. Most of these residents had experienced a fall within the last six months and were on FRIDs. The resident bedroom was identified as the most frequent location for falls among the majority of residents. The majority of residents in this facility had cognitive impairment and dementia. It was clear that 54 (77%) and 13 (18%) residents were on polypharmacy and antipsychotics, respectively, for September 2021. Organizational managers and facility managers can use the dashboard information to identify facility-level risk factors and assist them with mandatory quality indicator reporting.

#### Nurse or Personal Carer

On the facility falls view, a stage 3 resident (ie, resident: 8010) was selected and drilled down to the resident falls view to analyze the resident-level risk factors, fall incident characteristics, and personalized recommendations to prevent and manage future fall risk (Figures 5 and 6). Risk factors: resident 8010 was in stage 3 (as of December 31, 2021) with delirium, dementia, and stroke (Figure 7). The resident was on polypharmacy and antipsychotic medications as of December 30, 2021. There was an increase in fall risk compared to December 30, 2021, which was generated by the embedded predictive model. The resident had five falls during the study period, of which, two were injurious. When considering the resident’s incidents timeline, four fall incidents had occurred in the last six months. The resident was on polypharmacy along with FRIDs (as of December 31, 2021) where he had a change in FRIDs in the last three days (ie, December 29-31, 2021). Characteristics of fall incidents: most of these incidents occurred during the afternoon in the resident’s bedroom including the hallway and caused injuries to the elbow, head, and knee. The most recent FRAT assessment was conducted in April 2021. Recommendations to prevent future falls: according to the recent FRAT, the identified strategies in managing fall risk were displayed along with prescriptive recommendations such as “regular monitoring,” “assessing environmental risk in toilet or bathroom and bedroom,” and “assessing FRIDs use.”

For a further detailed view of medication use, the resident’s medications in detail view (Figure 7) can be assessed. Moreover, carers can use the information provided in the recommendation panel when assessing the fall risk for the resident, based on the resident-level risk factors (eg, dementia), environmental risk factors (ie, bedroom), etc.

## Discussion

### Principal Findings

Our dashboard adapted and refined the dashboard development process outlined by Staron [10], with an emphasis on understanding the specific domain of residential aged care and their current work practices around fall prevention and management. This was achieved through continuous stakeholder discussions (ie, Stages A and B), quantitative data analysis on the extracted datasets, and reviewing the published literature. This work produces important new resources for the current state of falls prevention in RACFs along with the extent of evidence-based prevention strategies. The dashboard design stage was an iterative process that required close collaboration with the users and its stakeholders (ie, Stage B). This was the most important stage of the dashboard development as it identified the data presentation for dashboard views along with the quantitative measures to be included for fall prevention. To enhance the technology implementation process within the context of fall prevention and management, the four constructs of the integrated Promoting Action on Research Implementation in Health Services framework were followed throughout the dashboard development process.

The core innovation of this study comes from several novel aspects. The Royal Commission into Aged Care Quality and Safety has noted that the residential aged care sector has lagged behind other health care sectors in adopting and implementing technology [30]. It is also found that information systems and processes in these settings are both underdeveloped and inadequately integrated [31]. Furthermore, successfully connecting multiple disparate data sources in aged care is uncommon. This may be due to various factors, such as data being collected by different systems and third-party software vendors being inflexible in incorporating unique identifiers (ie, resident identifiers) across systems. Therefore, this study emphasizes the value of linking existing datasets and visualizing the linked information to support falls prevention. The resident falls view in the dashboard presents the linked data from four data sources and supports to identify resident-level risk factors, fall incident characteristics, and personalized recommendations for fall prevention. This allows users to access crucial information at a glance, rather than navigating across different systems, thus demonstrating significant added value in preventing falls.

The dashboard provides staff with easy access to synthesized, evidence-based recommendations for effective fall prevention strategies. In the dashboard, external evidence on fall prevention and management (eg, published prevention recommendations) has been aligned with current priorities and practices at RACFs (such as PH-FRATs). Current practices, such as the fall prevention strategies identified through PH-FRATs, were embedded, along with rule-based recommendations. Additionally, a separate recommendation panel was established to support decision-making around fall risk assessments.

Another novel aspect of the dashboard is the embedded predictive model. Predicting fall risk is challenging because of the numerous contributing factors [32,33], including medical conditions, medications, functional status, behavior, physiological aspects, and environmental conditions. Many of these factors, such as medication use, can vary over time [34]. The dynamic predictive model embedded in the dashboard is therefore an important part of the dashboard. It uses near real-time data and helps to classify the resident risk of falls on a daily basis. The incorporation of the predictive algorithm in the dashboard goes beyond what has been possible with existing fall risk assessments as it dynamically predicts the risk and links it to residents’ fall risk factors and personalized strategies. This provides timely and meaningful risk prediction along with evidence-based recommendations based on resident profile, to the caring staff to facilitate person-centered fall prevention and improve outcomes.

Finally, the dashboard is innovative because it adopts a holistic approach to managing falls in aged care residents by integrating multiple data sources and making this information easily accessible to different stakeholders including managers and carers. The granular resident-level data are aggregated to the facility and organizational levels, allowing users to navigate seamlessly between these levels with just a few clicks. This structure enables users to drill down from the organizational level to individual residents or vice versa, providing a comprehensive and detailed view of fall risk and prevention strategies.

### Challenges Faced and Strategies to Overcome

There were many challenges faced when developing this dashboard. Initially, the dashboard was developed using datasets that were extracted from the provider rather than directly connecting to the database. This leads to the integration process becoming more challenging unless the variables are in standard format as in the database. Moreover, our partner has changed its incident reporting system and daily medication management system within the development process. More robust processes were required to be implemented on the backend of the dashboard to overcome these challenges. The data acquisition layer discussed in dashboard architecture was important in this aspect. This layer acts as a data reservoir. Here, the initial data are extracted from original sources, stored in the original format, and then transformed into the data tables that the dashboard requires. This layer enabled the dashboard to be sustainable in the long run amid the underlying system changes.

The data model in the backend of the dashboard also plays a critical role in facilitating the above process. This is crucial as the measures were developed using the linkages between the tables. Adapting a star schema as much as possible within the dashboard backend has promoted several advantages within the study. Simplicity and ease of use of the data model during various stages of the development process can be noted. Visualization and embedding of analytical measures within the dashboard were simplified without complex data integration. This has streamlined the dashboard development during the co-design stage while accelerating the time of incorporating various views on fall prevention and management. Flexibility and scalability can be noted as another advantage where the schema was helpful in adapting the dashboard design to changing RACF requirements in managing falls while incorporating additional data fields and sources with minimum modifications to the existing structure. Therefore, overall, adapting the star schema as much as possible within the dashboard development process was an excellent choice due to its ability to incorporate changing user requirements in the context of fall management at RACFs.

Furthermore, for the daily calculation of changes in fall risk, this study uses R programming. This decision stems from PBI’s inability to retain and save daily risk calculations. To address this limitation, the designers have integrated R programming code into the Power Query Editor, enabling the storage of these risk calculations on a shared drive. The code automatically saves these calculations on a daily basis during the data refresh cycle, occurring every 24 hours. Subsequently, on a daily basis, these stored files (ie, previous-day risk calculations) are retrieved into the PBI backend to calculate the difference between current and previous risks at the resident level.

The integration of R programming into the dashboard backend has introduced a technical skills gap between the partner organization and the dashboard designers. Consequently, workshops were organized with the partner organization’s IT teams to provide training on R programming, bridging the gap in statistical analysis using R programming. Additionally, line-by-line code interpretation was incorporated into the embedded codes to facilitate a seamless transition during dashboard implementation.

### Implications for Policy, Practice, and Future Research

This study highlights the potential of using a fall prevention and management dashboard to enhance resident outcomes in aged care through the effective use of information systems. Health dashboards facilitate decision-making and are required to be used in conjunction with clinical judgment, staff education, training, and individualized tailored responses. Future policy and practice must ensure the interoperability of information systems in aged care, involve key stakeholders in development and implementation, and allocate appropriate funding for these processes. Future research should also focus on expanding the fall prediction dashboard and incorporating other care needs in residential aged-care facilities, such as hospitalization and wound management.

### Conclusions

In this study, a comprehensive process for developing a fall prevention and management dashboard with descriptive, predictive, and prescriptive analytics for RACFs was thoroughly discussed along with a sustainable architecture. The key focus was to provide a holistic approach to managing falls by linking existing data sources at the provider level. This highly applied and translational research demonstrated how EHRs can be used along with technology to deliver evidence-based information to aged care allied health workers and managers, to drive better aged care quality and safety.

## Appendix

Multimedia Appendix 1 Supplementary tables.

Multimedia Appendix 2 Dashboard evolution within the design stage.

## Abbreviations

DAX: Data Analysis Expression
EHR: electronic health record
FRAT: falls risk assessment tool
FRID: fall risk–increasing drug
PBI: Power BI
PH-FRAT: Peninsula Health Falls Risk Assessment Tool
RACF: residential aged care facility

## Data Availability

The datasets generated or analyzed during this study are not publicly available due to the privacy of the individuals who participated in the study but are available from the corresponding author upon reasonable request.
