# Supplementary material for: Development of a Predictive Dashboard With Prescriptive Decision Support for Falls Prevention in Residential Aged Care: User-Centered Design Approach
Source: JMIR Aging. 2025 Apr 7;8:e63609. doi: 10.2196/63609 (PMC12012402; doi:10.2196/63609)
Supplement: Multimedia Appendix 1 [file aging_v8i1e63609_app1.docx]

**Table S1**: Datasets utilised in design stage of the dashboard.

| Dataset | Details | Time period | Variables extracted | Type of variable |
| --- | --- | --- | --- | --- |
| Profile dataset | All residents (i.e. All residents enrolled to a RACF) | Between 2021/01/01-2021/12/31 | Resident ID | Numeric |
|  |  |  | Gender | Categorical |
|  |  |  | Year of birth | Date |
|  |  |  | Facility ID | Numeric |
|  |  |  | Entry type | Categorical |
|  |  |  | Entry date | Date |
|  |  |  | Departure date | Date |
|  |  |  | Active status | Categorical |
|  |  |  | Health status (free text) | Free text |
| Incident dataset | All falls and pressure injuries | Between 2021/01/01-2021/12/31 | Resident ID | Numeric |
|  |  |  | Date of incident | Date |
|  |  |  | Time of incident | Time |
|  |  |  | Type of incident | Categorical |
|  |  |  | Location of incident | Categorical |
|  |  |  | Body regions injured | Categorical |
|  |  |  | Hospitalisation required | Categorical |
| Daily medication administration | All daily medication administration records | Between 2021/01/01-2021/12/31 | Resident ID | Numeric |
|  |  |  | Date of administration | Date |
|  |  |  | Time of administration | Time |
|  |  |  | Drug name | Free text |
|  |  |  | Administration status | Categorical |
|  |  |  | Dosage | Numeric |
|  |  |  | Self-administration status | Categorical |
|  |  |  |  |  |
| PH FRATs | All falls risk assessments | Between 2021/01/01-2021/12/31 | Resident ID | Numeric |
|  |  |  | Date completed | Date |
|  |  |  | Mobility status | Categorical |
|  |  |  | Cognitive status | Categorical |
|  |  |  | Strategies managing falls risk | Free text |
|  |  |  | I am supported to maintain my social relationships and connections with the community | Five point Likert scale |
|  |  |  | I am comfortable lodging complaints with confidence that the appropriate action will be taken | Five point Likert scale |

**Table S2**: Admission related information from resident profile dataset.

| Admission related information ^a^ | |
| --- | --- |
| **Number of admissions** | **N = 3686***^1^* |
| Number of facilities | 24 |
| Facility size | 124 (86, 160) |
| Entry date | 1977-07-22 to 2021-12-31 |
| Length of stay | 373 (41, 1,266) |
| Entry type |  |
| Permanent | 2,622 (71%) |
| Respite | 1,060 (29%) |
| Interim care | 4 (0.1%) |
| Departure reason |  |
| Active | 2046 (55%) |
| Deceased | 632 (17%) |
| Other | 487 (13%) |
| Wait return to family | 334 (9%) |
| To hostel | 66 (2%) |
| To hospital | 63 (2%) |
| To other aged care facility | 58 (2%) |
| This table accounts for unique admission episodes. Some residents may have multiple admissions to the same or different facility. *^1^* n (%); Range; Median (IQR) | |

**Table S3**: Resident characteristics from resident profile dataset, FRAT dataset and daily medication administration data.

| Variable | Overall, N = 3,057*^1^* |
| --- | --- |
| **Resident profile dataset** | |
| Gender |  |
| Female | 2,030 (66%) |
| Male | 1,025 (34%) |
| Unknown | 2 |
| Age ^a^ | 85 (79, 90) |
| Country of Birth |  |
| Australia | 1,944 (72%) |
| Other countries | 765 (28%) |
| Unknown | 348 |
| Total length of stay | 665 (144, 1,479) |
| Number of admissions |  |
| Once | 2,500 (82%) |
| More than once | 557 (18%) |
| Entry type ^a^ |  |
| Permanent | 2,104 (69%) |
| Respite | 950 (31%) |
| Interim care | 3 (<0.1%) |
| **Health status** | |
| Circulatory disease, any | 2,571 (84%) |
| Cerebrovascular accident | 714 (23%) |
| Endocrine, any | 1,134 (37%) |
| Diabetes | 796 (26%) |
| Thyroid disorder | 338 (11%) |
| Chronic respiratory disease | 537 (18%) |
| Neoplasms/cancer | 849 (28%) |
| Dementia | 1,437 (47%) |
| Parkinson’s disease | 153 (5.0%) |
| Depression, mood & affective disorders | 1,283 (42%) |
| Anxiety & stress-related disorders | 932 (30%) |
| Peptic Ulcer Disease (PUD) & (Gastroesophageal Reflux Disease) GORD | 918 (30%) |
| Renal disease | 541 (18%) |
| Arthritis | 1,615 (53%) |
| Osteoporosis | 813 (27%) |
| Gout | 203 (6.6%) |
| History of fracture | 1,022 (33%) |
| Hearing impairment | 644 (21%) |
| Visual impairment | 479 (16%) |
| Delirium | 367 (12%) |
| Cognitive impairment | 1,158 (38%) |
| **PH-FRATs** | |
| Mobility - mobility status unknown or appears unsafe/impulsive/forgets gait aid ^b^ |  |
| No | 501 (26%) |
| Yes | 1,455 (74%) |
| Unknown | 1,101 |
| Behaviours - agitation/confusion/disorientation/difficulty following instructions/non-compliant ^b^ |  |
| No | 700 (36%) |
| Yes | 1,256 (64%) |
| Unknown | 1,101 |
| ADLs - risk taking behaviours or unsafe use of equipment/inappropriate footwear or clothing ^b^ |  |
| No | 1,193 (61%) |
| Yes | 763 (39%) |
| Unknown | 1,101 |
| **Daily medication administration** | |
| More than 5 medications ^c^ |  |
| No | 856 (28%) |
| Yes | 2,201 (72%) |
| More than 7 medications ^c^ |  |
| No | 1,302 (43%) |
| Yes | 1,755 (57%) |
| More than 10 medications ^c^ |  |
| No | 2,053 (67%) |
| Yes | 1,004 (33%) |
| Usage of FRIDs ^c^ |  |
| No | 688 (23%) |
| Yes | 2,369 (77%) |

This table includes all resident-specific admission episodes. For residents with multiple admission episodes, the data are aggregated and analysed.

| *^1^* n (%); Median (IQR) |
| --- |
| *^a^ First admission considered during the study period*  *^b^ From most latest assessment conducted*  ^c^ *At least once during the stay* |

**Table S4**: Rule-based recommendations included in the *resident falls* view.

| Variable | Status/Rule | Subcategory of recommendation | Recommendation |
| --- | --- | --- | --- |
| FRIDs use | Yes | Assess for fall history | —Assess for fall history and the risk of falls before prescribing potential fall risk increasing drugs (FRIDs) to older adults |
|  |  | Identify FRIDs and their effects on the resident | —Use a validated, structured screening and assessment tool to identify FRIDs when performing a medication review |
|  |  | Ensure correct dose, frequency and type | —Ensure resident is taking medications as prescribed (i.e., in correct dose/frequency/type). |
|  |  | Conduct a medication review | —Include medication review and appropriate deprescribing of FRIDs as a part of the multifactorial falls prevention intervention |
|  |  | Deprescribing of FRIDs through a medication review | —In long-term care residents, if multifactorial intervention cannot be conducted due to limited resources, the falls prevention strategy should still always include deprescribing of FRIDs |
|  |  | Education for falls prevention and management | Some useful links:  1. Falls prevention in older adults Assessment and management: https://www.racgp.org.au/afp/2012/december/falls-prevention  2. Reducing fall risk for patients on sedating medications: https://www.cec.health.nsw.gov.au/__data/assets/pdf_file/0012/399639/Reducing-Fall-Risk-for-Patients-on-Sedating-Medications.pdf 3. Medication-related Falls Risk Assessment Tool (MFRAT):https://www.nswtag.org.au/wp-content/uploads/2020/11/NSW-TAG-8.2_Med-related-Falls-Risk-Assessment-ToolMFRAT.pdf 4. Medicines: balancing intended benefits and increased falls risk:https://www.hqsc.govt.nz/assets/Our-work/System-safety/Reducing-harm/Falls/Projects/Topic-EIGHT-June-2020.pdf  5. Medication and Falls: https://www.physio-pedia.com/Medication_and_Falls |
| Location | Resident's room | (Re-)assessing environmental risk in resident's room | —Recommend (re-)assessing environmental risk of falls for resident's room. |
|  |  | Ensure enough lighting in bedroom | —Ensure curtain properly adjusted (e.g., pull curtains back/down to increase lighting when it is dim/glaring during daytime). |
|  |  |  | —Install light bulbs with higher wattage. |
|  |  |  | —Use glow-in-the-dark light switches. |
|  |  |  | —Use night light. |
|  |  |  | —Install lights in dark closets. |
|  |  |  | —Store flashlight in an easy-to-find place in case a power outage. |
|  |  |  | —Ensure light switch easy to reach. |
|  |  | Ensure bedroom decluttered | —Clean room regularly to keep neat and tidy. |
|  |  |  | —Remove all clutters (e.g., newspaper, books and plant pots). |
|  |  |  | —Keep walkway clean. |
|  |  |  | —Remove loose rugs/carpets or have them secured with double-sided tape or repaired. |
|  |  |  | —Tuck electric cords under furniture or around skirting boards and tape them down. |
|  |  |  | —Make sure quilts, bedspreads and curtains do not fall across the floor. |
|  |  | Ensure furniture and bedroom design low-fall-risk | —Review furniture placement to optimise space. |
|  |  |  | —Use simple furniture arrangement. |
|  |  |  | —Ensure edges of furniture (e.g., tables, chairs and benches) smooth. |
|  |  |  | —Have furniture a contrasting colour to the walls and floor (e.g., furniture in dark colour and wall in light colour). |
|  |  |  | —Keep furniture in consistent places. |
|  |  |  | —Remove light, unstable or low pieces of furniture. |
|  |  |  | —Use chair with arms. |
|  |  |  | —Avoid heavily patterned floor coverings. |
|  |  |  | —Ensure doorsills are no more than one inch high. |
|  |  | Reduce fall risk in bed | —Ensure bed height appropriately adjusted so easy to climb and get out of. |
|  |  |  | —Recommend adjusting bed height to lowest at night. |
|  |  |  | —Check if instalment of bed rail, sensor and lolo/crash mat needed. |
|  |  |  | —Check if walking aids (e.g., 4-wheel-walker/walking stick) & caller within reach when residents are in bed. |
|  |  |  | —Place a table next to bed with important items (e.g., water, eyeglasses, keys, tissues and telephone) at night. |
|  |  |  | —Place a bedside lamp easy to turn on/off. |
|  | Shared indoor area | (Re-)assessing environmental risk in shared indoor area | —Recommend (re-)assessing environmental risk of falls for shared indoor area. |
|  |  | Encourage residents to familiarise with indoor communal space | —Encourage attendance to social activities, e.g., exercise/physio class. |
|  |  |  | —Encourage spending time in communal spaces, e.g., eating in dining room. |
|  |  |  | —Redirect residents when they lost their way. |
|  |  |  | —Ensure residents familiar with emergency escape route to avoid falls in case of emergency (e.g., fire). |
|  |  |  | —Add signage to doors and walkways to help residents find their way around |
|  |  | Extra cautiousness on stairs | —Install handrail next to stairs. |
|  |  |  | —Alert sign of fall risk next to stair. |
|  |  |  | —Use contrasting-coloured non-slip tapes on stairs to highlight edges and prevent slipping. |
|  |  |  | —Avoid organisation of activities on the second floor/lower ground. |
|  |  |  | —Encourage staff companion when leave ground floor. |
|  |  |  | —Caller installed next to stairs. |
|  |  | Ensure enough lighting in shared indoor space | —Leave lights on at night in the passageway and other frequently used places. |
|  |  |  | —Install two-way light switches on stairs or in corridors/rooms where there is more than one entry. |
|  |  |  | —Remove anything casting shadows on pathways. |
|  |  |  | —Install motion-sensor light. |
|  |  | Ensure floor low-risk-of-fall | —Don’t polish floor. |
|  |  |  | —Avoid waxing floor. |
|  |  |  | —Clean food and mop up spills immediately in communal dining areas. |
|  |  |  | —Remove, or mark, even small changes in floor level with contrasting colour or tape so they are easily seen |
|  | Bathroom/toilet | (Re-)assessing environmental risk in bathroom | —Recommend (re-)assessing environmental risk of falls for resident's bathroom/toilet. |
|  |  | Ensure grab rails/bars installed | —Install bathroom rails/grab bars for the shower/bathtub/toilet. |
|  |  |  | —Install darker handrails on light coloured walls. |
|  |  |  | —Ensure grab rails/bars with residents’ easy reach when they use toilet/shower. |
|  |  | Avoid slippery floor | —Install contrasting-coloured non-slip mats in bathtub/shower floor. |
|  |  |  | —Cover the whole toilet floor with non-slip material (e.g., epoxy coating designed to prevent skidding and slipping). |
|  |  |  | —Use anti-slip tape/mats on toilet floor. |
|  |  |  | —Apply contrasting coloured tape on the edge of the tub. |
|  |  |  | —Install a sturdy and contrasting-coloured shower chair. |
|  |  |  | —Recommend sitting down when showering and use a hand-held shower nozzle. |
|  |  | Ensure enough lighting | —Place night lights in the bathroom and the hallway to the bathroom. |
|  |  |  | —Light the area adequately |
|  |  | Have an accessible emergency system close by | —Ensure caller installed, workable and within reach in bathroom. |
|  |  |  | —Ensure residents know where the callers are in the bathroom. |
|  |  | Bathroom design | —Bathroom and toilet doors can open outwards. |
|  |  |  | —Have soap holders removed and they will not hit residents when they fall. |
|  |  |  | —Ensure low/no shower hob. |
|  | Garden/outdoor area | (Re-)assess environmental risk in Garden/outdoor area | —Recommend (re-)assessing environmental risk of falls for garden. |
|  |  | Remove trip hazards in garden/outdoor areas | —Keep paths clean and clear and with good drainage. |
|  |  |  | —Remove all objects in baths that might cause falls. |
|  |  |  | —Repair path if broken, uneven and cracked. |
|  |  |  | —Eliminate mosses/fungi/algae/slime/fallen leaves from garden/outdoor path. |
|  |  |  | —Don’t leave things lying around the garden (e.g., roll up the hose). |
|  |  | Ensure enough lighting in outdoor area | —Ensure outdoor space, particularly frequently used pathway, well-lit at night. |
|  |  | Have handrail/signage | —Have handrail/signage next to steps/stairs. |
|  |  | Wear safe shoes | —Recommend residents to wear safe shoes. |
|  |  | Check before moving | —Recommend residents to check before moving when there are fast moving objects nearby (e.g., pet/child/bicycle). |
| Mobility issues | Yes | Conduct a FRAT assessment | —Conduct a FRAT assessment |
|  |  | Recommendations for device use | —Consult a physiotherapist about whether a walking aid (e.g., walking stick or 4-wheel frame) is needed to  increase steadiness and confidence to walk more. |
|  |  |  | —Consult a doctor, physiotherapist or occupational therapist about whether hip and/or limb protectors are needed. |
|  |  | Regularly check the quality of device to ensure them workable | —Walking frame has been adjusted to an appropriate height, within reach and have quality and condition monitored. |
|  |  |  | —Check and clean glasses regularly. |
|  |  |  | —Ensure call bell is within reach and function checked. |
|  |  |  | —Monitor footwear/grip socks for suitability, ensuring residents are wearing appropriate, well fitting, non-slip  footwear/socks. |
|  |  |  | —Ensure sensor mat/door sensor (if used) is functioning, activated and placed at bedside. |
|  |  |  | —Ensure crash mats (if used) placed both sides of bed. |
| Risk stage | Stage 4 |  | — Close supervision and care is needed; staff to assist when residents move/transfer.  —A Falls Risk Assessment should be conducted. |
|  | Stage 3 |  | —Regularly check the residents to ensure he/she is safe.  —Please check whether a Falls Risk Assessment has been conducted recently (within the last 3 months). If not please perform. |
|  | Stage 2 |  | —Check residents 2 times/day |
|  | Stage 1 |  | —Encourage residents to keep active (e.g., Tai Chi/dancing/group exercise). |
