# Supplementary figures and images for: Development of a Predictive Dashboard With Prescriptive Decision Support for Falls Prevention in Residential Aged Care: User-Centered Design Approach
Source: JMIR Aging. 2025 Apr 7;8:e63609. doi: 10.2196/63609 (PMC12012402; doi:10.2196/63609)

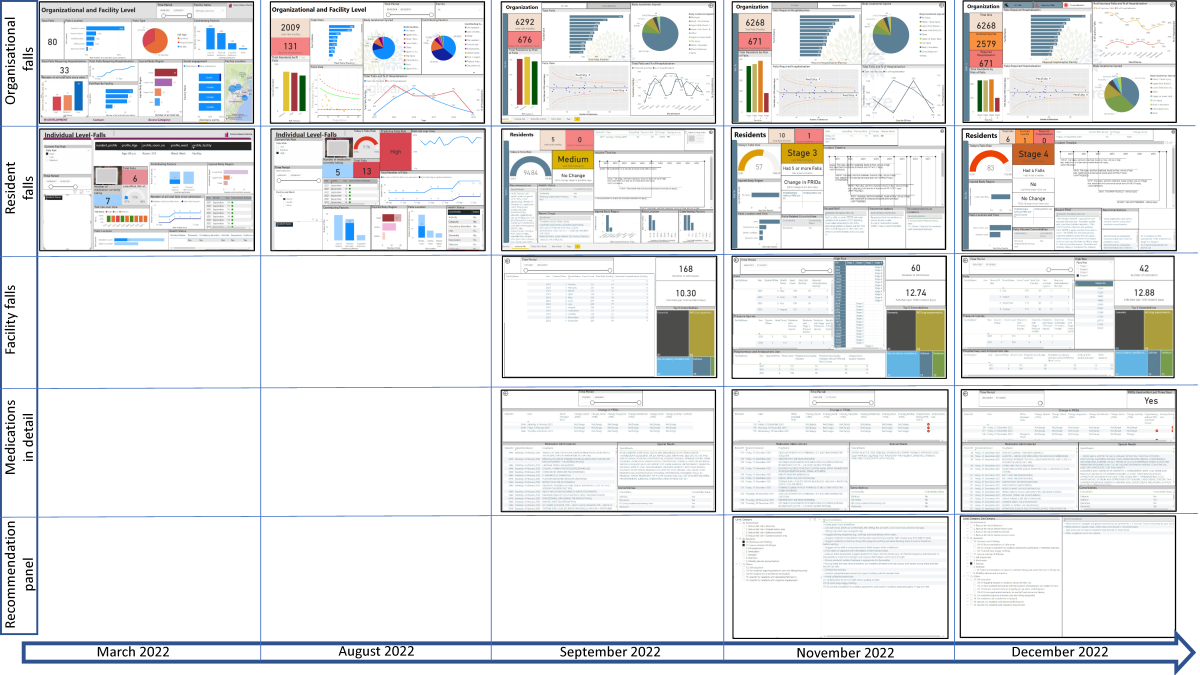

Supplement: Multimedia Appendix 2 [file aging_v8i1e63609_app2.png]
